# Comorbidities in Male Patients With Lichen Sclerosus: A Case-Control Study

**DOI:** 10.1097/LGT.0000000000000769

**Published:** 2023-09-20

**Authors:** Niina K. Hieta, Marjut A.M. Haataja, Lotta Tapana

**Affiliations:** 1 Department of Dermatology, Turku University Hospital and University of Turku, Turku, Finland; 2 Department of Obstetrics and Gynecology, Turku University Hospital and University of Turku, Turku, Finland; 3 Auria Clinical Informatics, Wellbeing Services County of Southwest Finland, Turku, Finland

**Keywords:** lichen sclerosus, case-control study, penile carcinoma, cardiovascular diseases, lichen planus, atopic dermatitis, comorbidities

## Abstract

**Objective:**

Lichen sclerosus (LS) is a chronic inflammatory skin disease. In male patients, it usually involves the glans penis and foreskin and can cause phimosis or meatal stenosis. The aim of this cross-sectional case-control study was to identify clinically important comorbidities in male patients with LS.

**Materials and Methods:**

By searching Turku University Hospital electronic health records, the authors identified 630 male patients diagnosed with LS between 2004 and 2020. To investigate possible comorbidities, the authors compared this patient group to a 10-fold larger control group.

**Results:**

The incidence of LS increased during the study period, from 5 to 27.5 per 100,000 men. Patients were most often diagnosed at 21 to 25 years of age. Patients with LS exhibited markedly increased risks of penile carcinoma (odds ratio [OR], 81.0; 95% CI = 10.82–3516.7; *p* < .001) and carcinoma in situ of the penis (OR = 60.5; 95% CI = 7.32–2738.9; *p* < .001). Patients also more commonly exhibited lichen planus (OR = 16.8; 95% CI = 8.97–32.39; *p* < .001), psoriasis (OR = 3.3; 95% CI = 1.80–5.70; *p* = .004), angina pectoris (OR = 1.8; 95% CI = 1.10–2.81; *p* = .013), obesity (OR = 2.6; 95% CI = 1.72–3.77; *p* < .001), type 2 diabetes (OR = 2.3; 95% CI = 1.74–3.09; *p* < .001), and hypertension (OR = 1.9; 95% CI = 1.53–2.37; *p* < .001). The most commonly performed urological procedures were operation for phimosis, uroflowmetry, and ultrasound measurement of residual urine.

**Conclusions:**

Genital malignancies, other dermatological conditions, and diseases related to metabolic syndrome should be considered when treating patients with LS.

Lichen sclerosus (LS) is a chronic inflammatory skin disease that mainly occurs on genital skin. In male patients, it usually involves the glans penis and foreskin and can cause phimosis or meatal stenosis. Among women, LS has been linked to autoimmune conditions, cardiovascular diseases, gastrointestinal morbidities, and urogynecological malignancies and disorders.^1–3^ In men, LS is a risk factor for penile squamous cell carcinoma and its precursors.^1^ It has been suggested that LS may develop after urological procedures, such as radical prostatectomy for prostate cancer.^4^ It has also been speculated that sexually transmitted diseases, especially human papillomavirus (HPV), may play a role in the pathogenesis of LS.^5^ Previous studies have suggested that LS may be linked to atopic diseases in male patients^6,7^ and to lichen planus (LP) and psoriasis in female patients.^3^

Diseases related to metabolic syndrome and cardiovascular diseases are more common in female patients with LS,^3^ but conflicting data have been reported from studies of male patients, possibly because many studies include low numbers of patients.^8–12^ While autoimmune diseases have been linked to LS in female patients, this does not seem to be the case among male patients with LS.^1^ Many of the investigations of LS-associated comorbidities in male patients have been small studies, without a control group.

In the present study, we investigated diseases that have been previously described in male and female patents with LS, as possible comorbidities. These diseases included penile cancer and in situ carcinoma of penis. We also examined urethral stricture, phimosis, and balanoposthitis because they are typical features of male LS. Moreover, because LS may develop after urological procedures, we investigated prostate cancer and hyperplasia of the prostate, which are often treated with transurethral procedures. Autoimmune diseases seem to be rare among male patients with LS. We chose to study hypothyroidism, which is the most common autoimmune disease in female patients with LS. We also investigated sleep apnea, which is related to metabolic disease, especially obesity but has not previously been studied in patients with LS. Based on our own clinical observation that patients often have problems with venous circulation of the lower limbs, we also examined phlebitis, thrombophlebitis, and chronic peripheral venous insufficiency. We also investigated the urological procedures performed in LS patients to increase our understanding of the disease burden of LS.

## METHODS

This study included male patients who had been diagnosed with LS during the period from January 1, 2004, to December 31, 2020, at Turku University Hospital. Patients were identified either by the *International Classification of Diseases*, *Tenth Edition* (*ICD-10*) diagnosis code L90.0, or leukoplakia of the penis/balanitis xerotica obliterans (N48.0), or from the pathology information system (QPATI) using the organ diagnosis search term “lichen sclerosus et atrophicus.” The patients were diagnosed and treated at the departments of dermatology or urology, which traditionally use different *ICD-10* codes. The collected data included the patient's sex and age at the time of diagnosis, QPATI diagnosis, and all of the patient's *ICD-10* diagnoses. We also collected data about the urological procedures performed in LS patients, which were classified according to the Nordic Classification of Surgical Procedures.

**TABLE 1:** Urological Procedures Performed Among 630 Male Patients With LS

Procedure	Total no. procedures	Total no. patients (% of all patients with LS)	No. patients with 1 procedure (% of all patients with LS)	No. patients with 2 procedures (% of all patients with LS)	No. patients with 3 or more procedures (% of all patients with LS)
Operation for phimosis	415	401 (63.7)	387 (61.4)	14 (2.2)	0 (0)
Uroflowmetry	405	185 (29.4)	74 (11.7)	62 (9.8)	49 (7.8)
Ultrasound measurement of residual urine	373	173 (27.5)	73 (11.6)	55 (8.7)	45 (7.1)
Cystoscopy	137	78 (12.4)	47 (7.4)	18 (2.9)	13 (2.1)
Penis blockade	57	56 (8.9)	55 (8.7)	1 (0.2)	0 (0)
Urinary tract ultrasound examination	53	38 (6.0)	32 (5.1)	2 (0.3)	4 (0.6)
Dilatation of urethra	46	37 (5.9)	31 (4.9)	3 (0.5)	3 (0.5)
Rectal ultrasound examination of prostate	44	34 (5.4)	27 (4.3)	5 (0.8)	2 (0.3)
Ultrasound examination of scrotum	39	33 (5.2)	29 (4.6)	2 (0.3)	2 (0.3)
Catheterization of bladder	36	26 (4.1)	20 (3.2)	2 (0.3)	4 (0.6)
Internal urethrotomy	34	26 (4.1)	19 (3.0)	6 (1.0)	1 (0.2)
CT of urinary organs	29	14 (2.2)	9 (1.4)	3 (0.5)	2 (0.3)
Urinary tract x-ray examination without contrast	28	7 (1.1)	3 (0.5)	0 (0)	4 (0.6)
Teaching patient the catheterization of bladder	25	20 (3.2)	17 (2.7)	2 (0.3)	1 (0.2)
Biopsy of penis	23	19 (3.0)	17 (2.7)	1 (0.2)	1 (0.2)
Meatoplasty of urethra	17	16 (2.5)	15 (2.4)	1 (0.2)	0 (0)
Transurethral resection of prostate	16	14 (2.2)	12 (1.9)	2 (0.3)	0 (0)

For each identified patient, we randomly selected 10 age- and sex-matched controls with no diagnosis of LS from the hospital's electronic health records. The electronic health records contain clinical data for all patients who visited public hospitals in the hospital district from 2004 onward. The hospital district includes more than 470,000 residents, and university hospital also serves as a tertiary clinic for 2 other hospital districts. More than 200,000 people use the services of university hospital every year. This study also included patients from other hospital districts who were referred to university hospital for any reason and whose health records were available. The proportions of patients from other hospital districts were similar between the patient and control groups. We studied urogenital diseases and malignancies, dermatological and atopic diseases, sexually transmitted and other genital infections, diseases related to metabolic syndrome and cardiovascular diseases, and thyroid diseases. This study was approved by the hospital district, approval number T05/037/21. According to national legislation, informed consents are not required for studies based on patient records.

### Statistical Analysis

Continuous variables were described using mean and SD if normally distributed, or median and lower and upper quartiles (Q1–Q3) if nonnormally distributed. Discrete variables were presented using observed frequency and proportion. We analyzed the association of LS with other diseases using Fisher exact test. The results are presented as odds ratios (ORs), together with 95% CIs and *p* values. The Benjamini-Hochberg method was used to adjust *p* values for multiple comparisons. Statistical analyses were performed, and tables, figures, and listings were produced using R version 3.6.3 (R Core Team, 2018) with RStudio Server in secure operating environment.

## RESULTS

### Clinical Features

**FIGURE 1:**
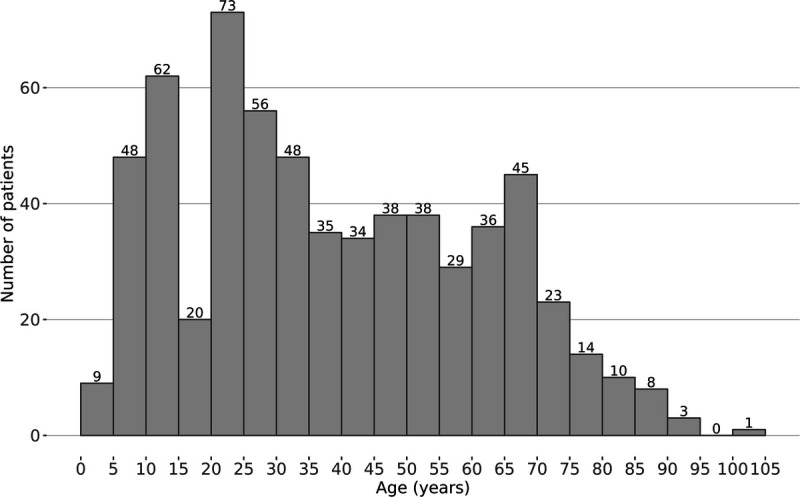
Incidence of lichen sclerosus in 630 men during 2004–2020 according to age, in 5-year increments.

We identified a total of 630 male patients with a diagnosis of lichen sclerosus (L90.0 or the corresponding QPATI term) or leukoplakia of the penis/balanitis xerotica obliterans (N48.0). Of these patients, 290 were identified based on histological diagnosis, 462 based on the *ICD-10* classification in electronic health records, and 122 based on both histological diagnosis and *ICD-10* classification. The mean patient age was 39.2 years (range, 2–101 years), and the median age was 35.0 years. There were 119 patients aged 2 to 15 years, and 144 patients 60 years or older. The patients in our study had most commonly been diagnosed at 21 to 25 years of age (see Figure 1).

**FIGURE 2:**
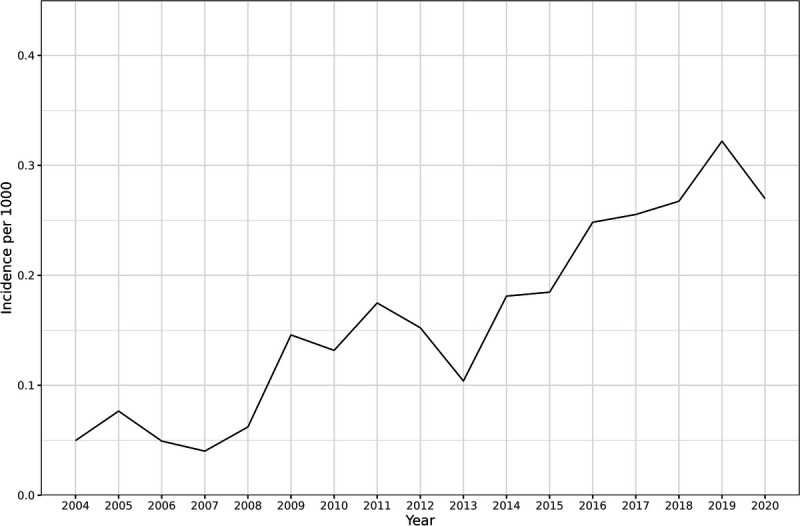
Incidence of lichen sclerosus in men during 2004–2020.

The incidence of LS increased during the study period (see Figure 2)—from 5 per 100,000 men in 2004, to 27.5 per 100,000 men in 2020, with the highest incidence of 32.5 per 100,000 men in 2019.

### Urogenital Diseases and Malignancies

Penile carcinoma was present in 8 patients with LS, but in only one of the controls (OR = 81.0; 95% CI = 7.32–2738.9) (see Table 1). The cases of penile cancer included 5 in the prepuce, 4 in the glans, 2 in the body of the penis, and 1 overlapping—with some patients having multiple penile carcinomas during the study period. Carcinoma in situ of the penis was diagnosed in 6 patients with LS and in 1 of the controls (OR = 60.5; 95% CI = 10.82–3516.7). Prostate cancer was more common among patients with LS (4.6%; OR = 1.7; 95% CI = 1.07–2.49), as well as hyperplasia of the prostate (10.5%; OR = 1.9; 95% CI = 1.45–2.57), urethral stricture (4.4%; OR = 10.4; 95% CI = 5.90–18.37), and phimosis, which is a typical feature of LS (53.7%; OR = 102.7; 95% CI = 77.07–138.48).

**TABLE 2:** Comorbidities in 630 Male Patients With LS and in Their 10-Fold Male Control Patients

Comorbidity	*ICD-10*	Prevalence in men with LS, % (n)	Prevalence in control population,% (n)	OR	95% CI	*p*
Urogenital diseases and malignancies						
Penile carcinoma	C60	1.3 (8)	0.016 (1)	81.0	10.82–3516.7	<.001
Carcinoma in situ of penis	D07.4	1.0 (6)	0.016 (1)	60.5	7.32–2738.9	<.001
Prostate cancer	C61	4.6 (29)	2.8 (178)	1.7	1.07–2.49	.019
Hyperplasia of the prostate	N40	10.5 (66)	5.7 (358)	1.9	1.45–2.57	<.001
Urethral stricture	N35	4.4 (28)	0.44 (28)	10.4	5.90–18.37	<.001
Phimosis	N47	53.7 (338)	1.1 (70)	102.7	77.07–138.48	<.001
Lichen planus, psoriasis, and atopic diseases						
Lichen planus	L43	4.6 (29)	0.29 (18)	16.8	8.97–32.39	<.001
Psoriasis	L40	2.9 (18)	0.89 (56)	3.3	1.80–5.70	.004
Atopic dermatitis	L20	4.1 (26)	1.7 (109)	2.4	1.52–3.81	.009
Asthma	J45	6.0 (38)	3.7 (236)	1.6	1.13–2.36	.008
Vasomotor and allergic rhinitis	J30	4.4 (28)	1.8 (116)	2.5	1.57–3.81	.004
Sexually transmitted and other genital infections						
Anogenital warts	A63	6.8 (43)	1.1 (67)	6.8	4.49–10.24	<.001
Chlamydial infection	A56	4.3 (27)	1.4 (86)	3.2	2.00–5.08	<.001
Anogenital herpes simplex infection	A60	2.2 (14)	0.38 (21)	6.8	3.18–14.09	<.001
Balanoposthitis	N48.1	15.7 (97)	0.89 (56)	20.3	14.26–29.05	<.001
Diseases related to metabolic syndrome and cardiovascular diseases						
Obesity	E66	5.7 (36)	2.3 (145)	2.6	1.72–3.77	<.001
Type 2 diabetes mellitus	E11	10.6 (67)	4.9 (306)	2.3	1.74–3.09	<.001
Sleep apnea	G47.3	11.5 (71)	5.9 (369)	2.0	1.54–2.68	<.001
Essential hypertension	I10	19.5 (123)	11.3 (709)	1.9	1.53–2.37	<.001
Disorders of lipoprotein metabolism	E78	8.7 (55)	4.6 (288)	2.0	1.45–2.71	<.001
Angina pectoris	I20	3.8 (24)	2.2 (136)	1.8	1.10–2.81	.013
Phlebitis and thrombophlebitis	I80	2.4 (15)	0.83 (52)	2.9	1.52–5.32	.030
Chronic peripheral venous insufficiency	I87.2	2.9 (18)	0.86 (54)	3.4	1.86–5.94	.004
Thyroid diseases						
Hypothyreosis	E03	2.2 (14)	0.85 (54)	2.6	1.34–4.83	.005

### Lichen Planus, Psoriasis, and Atopic Diseases

Among the patients with LS, 4.6% had LP (OR = 16.8; 95% CI = 8.97–32.39), 2.9% had psoriasis (OR = 3.3; 95% CI = 1.80–5.70), and 4.1% had atopic dermatitis (OR = 2.4; 95% CI = 1.52–3.81) (see Table 2). Other atopic diseases were also common, including asthma (6.0%; OR = 1.6; 95% CI = 1.13–2.36) and vasomotor and allergic rhinitis (4.4%; OR = 2.5; 95% CI = 1.57–3.81). The LS patients who were 2 to 15 years of age exhibited a 2.0-fold increased risk for atopic eczema; however, this finding was not statistically significant after statistical correction (95% CI = 0.87–4.05). This youngest age group also showed more common occurrence of 2 other atopic diseases, asthma (OR = 1.8; 95% CI = 0.95–3.33) and vasomotor and allergic rhinitis (OR = 2.8; 95% CI = 1.20–5.88), but again these results were not statistically significant.

### Sexually Transmitted and Other Genital Infections

Among patients with LS, 6.8% had anogenital (venereal) warts (OR = 6.8; 95% CI = 4.49–10.24), and 2.2% had anogenital herpes infection (OR = 6.8; 95% CI = 3.18–14.09). Chlamydial infection was present in 4.3% of patients with LS (OR = 3.2; 95% CI = 2.00–5.08), and balanoposthitis was present in 15.7% of patients (OR = 20.3; 95% CI = 14.26–29.05).

### Diseases Related to Metabolic Syndrome, Cardiovascular Diseases, and Thyroid Diseases

Patients with LS were more often diagnosed with obesity (5.7%; OR = 2.6; 95% CI = 1.72–3.77). Among patients with LS, type 2 diabetes was present in 10.6% (OR = 2.3; 95% CI = 1.74–3.09), sleep apnea in 11.5% (OR = 2.0; 95% CI = 1.54–2.68), essential hypertension in 19.5% (OR = 1.9; 95% CI = 1.53–2.37), and hypercholesterolemia in 8.7% (OR = 2.0; 95% CI = 1.45–2.71). Angina pectoris was diagnosed in 3.8% of patients with LS (OR = 1.8; 95% CI = 1.10–2.81). Among patients 60 years or older, the ORs for comorbidities were very similar to the ORs of all patients (data not shown). The only exception was obesity, for which the OR was even higher for patients 60 years or older (OR of 4.0, compared with 2.6 among all patients; 95% CI = 2.0695–7.2762).

Phlebitis or thrombophlebitis was present in 2.4% of patients with LS (OR = 2.9; 95% CI = 1.52–5.32), and chronic peripheral venous insufficiency in 2.9% (OR = 3.4; 95% CI = 1.86–5.94). Hypothyroidism was present in 2.2% of patients with LS (OR = 2.6; 95% CI = 1.34–4.83).

### Urological Procedures

We also studied the urological procedures performed in LS patients (see Table 2). The most common procedures were operation for phimosis (63.7% of patients), uroflowmetry (29.4% of patients), and ultrasound measurement of residual urine (27.5% of patients). Procedures suggestive of a tight urethra were common, including dilatation of urethra (5.9% of patients), internal urethrostomy (4.1% of patients), and meatoplasty of urethra (2.5% of patients).

## DISCUSSION

In our patients with LS, the mean age at the time of diagnosis was 39.2 years, which is in line with previous studies, where the mean age has ranged from 39.3 to 51.3 years.^6,8,12,13^ The patients in our study were most commonly diagnosed with LS at 21 to 25 years of age (see Figure 1). On the other hand, female patients with LS are most commonly diagnosed at 60 to 69 years of age.^14^ The reason for this difference is not known, but hormonal factors may play a role.

During our study period, the incidence of LS increased by more than 5-fold (see Figure 2). This increase may partly be due to more accurate diagnoses but may also partly indicate a true increase of incidence. Among female patients, the incidence of LS has increased from 14 per 100,000 woman-years in 2003 to 22 per 100,000 woman-years in 2010.^15^

While it is known that male patients with LS have an increased risk of genital malignancies, few studies have aimed to determine the exact degree of increased risk.^1,16^ Among the male patients with LS in our study, 1.3% also had penile carcinoma (OR = 81.0) and 0.95% had penile in situ carcinoma (OR = 60.5). As emphasized by Kravvas et al.,^17^ effective treatment of LS and penile intraepithelial neoplasia can strongly reduce the frequency of malignant changes.

Lichen sclerosus may lead to meatal stenosis. On the other hand, LS may be induced by local trauma, such that treatments involving transurethral procedures or catheterization might trigger LS. This could explain why patients with LS more commonly had prostate cancer (OR = 1.7) and hyperplasia of prostate (OR = 1.9).

The presently observed increased risk of atopic diseases among LS patients supports the findings of previous smaller studies. In one study of 35 adult male patients with LS, 34% of patients had an atopic disease.^6^ Another prior study reported that atopic eczema was more common among boys with LS.^7^ The LS patients in our study who were 2 to 15 years of age exhibited increased risks of atopic eczema (OR = 2.0), asthma (OR = 1.8), and vasomotor and allergic rhinitis (OR = 2.8); however, these results were not statistically significant.

In contrast to earlier studies, we also found that LS patients had an increased risk of psoriasis. Previous studies of male patients^18^ or of a combined group of male and female patients^8^ have not shown a connection between LS and psoriasis. In addition, our study is the first to demonstrate an increased risk of LP among male patients with LS. A previous study demonstrated that female patients with LS had increased risks of psoriasis and LP.^3^

Human papillomavirus DNA is found in a median of 22% of all LS cases (range, 0%–80%).^5^ Accordingly, our study showed an increased risk of anogenital warts (OR = 6.8) in male patients with LS. It is not yet known what possible role HPV might play in the pathogenesis of LS.

Notably, the increased number of sexually transmitted diseases (including chlamydial and gonorrheal infections) among patients in our study could be related to the fact that many patients were diagnosed in the venereal diseases outpatient clinic, with the diagnosis of LS not being their original reason for contacting the clinic. The increased risk for balanoposthitis among LS patients in our study (OR = 20.3) may reflect that balanoposthitis was the initial clinical diagnosis or that balanoposthitis might have been a complication of phimosis.

Patients with LS were more often diagnosed with obesity (5.7%; OR = 2.6). This is in line with previous findings. Hofer et al.^9^ showed a higher body mass index in male patients with LS (31.0 vs. 28.1 kg/m^2^ in controls). In a study of both male and female LS patients, Virgili et al.^8^ showed a body mass index of ≥25 kg/m^2^ in 51.2% of patients with LS, compared with in 35.6% of controls. Notably, obesity can complicate the treatment of LS, as the penis may be totally enclosed by the suprapubic fat pad and, after circumcision, by a pseudo-foreskin formed by forward movement of the skin of the penis shaft.^19^

Angina pectoris was diagnosed in 3.8% of patients with LS (OR = 1.8), which is in line with a previous finding that the OR for coronary artery disease was 1.88 among patients with LS.^9^ Diseases related to metabolic syndrome—including obesity, type 2 diabetes, hypercholesterolemia, and hypertension—were about twice as common in male patients with LS compared with in the control group. This is the first report to show increased risks of all of these diseases in the same study population, as well as an increased risk of sleep apnea.

We also found that patients with LS had increased risks of phlebitis and thrombophlebitis (OR = 2.9) and chronic peripheral venous insufficiency (OR = 3.2). The cause of chronic peripheral venous insufficiency is not known, but it may predispose to phlebitis and thrombophlebitis. It has been suggested that the development of microvascular disease may be associated with LS,^9^ and it may be speculated that LS could have a direct effect on the vascular endothelium in these venous diseases.

Hypothyroidism was diagnosed in 2.2% of LS patients (OR = 2.6). This rate is slightly lower than in previous studies, where thyroid disease or abnormal laboratory values for thyroid function have been observed in 3.4% to 12.5% of male patients with LS.^8,13,18,20,21^

We also investigated the numbers of surgical and diagnostic urological procedures. Almost two thirds of LS patients underwent operation for phimosis. Patients also commonly underwent procedures suggestive of a tight urethra—including dilatation of urethra, internal urethrostomy, and meatoplasty of urethra. The high numbers of procedures for uroflowmetry and ultrasound measurement of residual urine indicated that patients frequently had some degree of difficulty with urination. Notably, it is possible that these procedures were the cause of LS via koebnerization.

A limitation of this study was its retrospective method. Some diagnoses made in primary health care may not have been recorded in the hospital's electronic health care system. This would result in underreporting of some diagnoses; however, in this context, the relative abundance of diseases between patients with LS and controls should remain the same. In addition, it is possible that the patients who were referred to the hospital had a more severe disease than patients treated by general practitioners. This is also suggested by the high proportion of patients who underwent operations for phimosis (63.7%), compared with 37.7% to 50% of patients having circumcision as a treatment of LS in previous studies.^14,17^

Because of the study design, it was not possible to determine whether LS was diagnosed before, after, or at the same time as the comorbidities. Therefore, based on this study, it is not possible to conclude whether LS is a cause or result of another morbidity or whether they derive from the same predisposing factor. The same is true for procedures, where it cannot be determined whether a diagnostic procedure (e.g., uroflowmetry) was performed due to difficulties in urination caused by LS, or rather whether it preceded LS and potentially caused LS through koebnerization.

## CONCLUSIONS

Penile carcinoma, diseases related to cardiovascular diseases and metabolic syndrome, LP, and atopic diseases should be considered possible comorbidities in male patients with LS. Our results suggest that the incidence of LS among men may be increasing. When patients undergo diagnostic procedures involving the urethra, the possibility of LS should be kept in mind if new relevant symptoms appear.
